# The Effect of Rosmarinic Acid on Apoptosis and nNOS Immunoreactivity Following Intrahippocampal Kainic Acid Injections in Rats

**DOI:** 10.32598/bcn.9.10.340

**Published:** 2020-01-01

**Authors:** Safoura Khamse, Seyed Shahabeddin Sadr, Mehrdad Roghani, Mina Rashvand, Maryam Mohammadian, Narges Marefati, Elham Harati, Fatemeh Ebrahimi

**Affiliations:** 1. Department of Physiology, School of Medicine, Tehran University of Medical Sciences, Tehran, Iran.; 2. Electrophysiology Research Center, Neuroscience Institute, Tehran University of Medical Sciences, Tehran, Iran.; 3. Neurophysiology Research Center, Shahed University, Tehran, Iran.; 4. Department of Physiology, School of Medicine, Kermanshah University of Medical Sciences, Kermanshah, Iran.

**Keywords:** Kainic acid, Rosmarinic acid, nNOS-positive neurons, TUNEL-positive cells, Mitogen-Activated Protein Kinase (MAPK) and Cyclooxygenase-2 (COX-2) immunoreactivity

## Abstract

**Introduction:**

Kainic Acid (KA) is an ionotropic glutamate receptor agonist. KA can induce neuronal overactivity and excitotoxicity. Rosmarinic Acid (RA) is a natural polyphenolic compound with antioxidant, anti-apoptotic, anti-neurodegenerative, and anti-inflammatory properties. This study aimed to assess the effect of RA on apoptosis, nNOS-positive neurons number, as well as Mitogen-Activated Protein Kinase (MAPK) and Cyclooxygenase-2 (COX-2) immunoreactivity, following intrahippocampal Kainic acid injection in rats.

**Methods:**

The study rats were randomly assigned to three groups of sham, KA (KA was injected into the right side of the hippocampus) and KA+RA (a dose of 10 mg/kg/day through a gavage needle for one week before KA injection). Then, histopathological changes, including apoptosis [Terminal Deoxynucleotidyl Transferase (TdT) dUTP Nick-End Labeling (TUNEL) assay], nNOS-positive neurons number, as well as COX-2 and MAPK immunoreactivity were evaluated in the hippocampus.

**Results:**

In the RA pretreated group, nNOS-positive neurons and TUNEL-positive cells were significantly reduced compared to the KA group (P<0.05). COX-2and MAPK immunoreactivity demonstrated no significant changes compared to the KA group. They indicated a significant higher reactivity for COX-2 (P<0.01) and MAPK (P<0.005) versus the sham group.

**Conclusion:**

RA had neuroprotective effects, compared to KA, through reduced apoptosis and nNOS-positive neurons, but not MAPK and COX-2.

## Highlights

- Kainic Acid (KA) is an ionotropic glutamate receptor agonist, which can induce neuronal overactivity and excitotoxicity.
- The immunohistochemical results suggested that KA group had a significantly higher number of nNOS-positive neurons.
- RA had neuroprotective effects, compared to KA, through reduced apoptosis and nNOS-positive neurons, but not MAPK and COX-2.

## Plain Language Summary

The Kainic Acid KA-induced seizure model is widely used as a standard model of human temporal lobe epilepsy. As a structural analog of glutamate, KA activates excitatory amino acid receptors and triggers neuronal membrane depolarization and increases calcium influx through voltage-dependent calcium channel opened by membrane depolarization. Compounds like RA could reduce DNA damage through their scavenging ability. It suggests a neuroprotective effect for this compound, which can prevent and manage various neurological disorders.RA could prevent Kainic Acid-induced apoptotic cell death. Besides, RA exerts a protective effect on astrocytes, as demonstrated by their increased viability and decrease. The present study aimed to assess the effect of RA on apoptosis, nNOS-positive neurons number, and COX-2 and MAPK immunoreactivity, following intrahippocampal KA in rats.

## 1. Introduction

Kainic Acid (KA) is a glutamate analog with neuronal overactivity and excitotoxicity ( Hsieh et al., 2011) by inducing vigorous depolarizations leading to cell death. KA is sometimes used for modeling the temporal lobe epilepsy ( Levesque & Avoli, 2013). An unrestricted wide spectrum of neuropathological changes can be resulted from the acute and sub-acute forms of activity due to KA. Its induction ability of status epilepticus is associated with apoptotic and necrotic cell death ( Swamy, Yusof, Sirajudeen, Mustapha, & Govindasamy, 2011). KA also enhances Mitogen-Activated Protein Kinase (MAPK) and Cyclooxygenase-2 (COX-2) expression ( Hsieh et al., 2011).

Labiatae family Plants such as perilla frutescens, mint, sage, oregano, perilla, and sweet basil ( Scheckel, Degner, & Romagnolo, 2008) have medical uses for infection, inflammation, depression, indigestion, weakness, memory enhancement, circulation improvement, and fragile blood vessels strengthening in traditional medicine. These plants have several compounds with various beneficial effects. These effects are attributed to their phenolic compounds, and especially Rosmarinic Acid (RA). RA has various biological and anti-pathological functions as astringent, anti-oxidant, anti-inflammatory, anti-bacterial, anti-mutagen, anti-cholinesterase, anti-tumor, hepatoprotective, and cardioprotective properties. Its anti-inflammatory activity can be observed by the inhibition of lipoxygenases and cyclooxygenases ( Tepe, 2008). Its anti-oxidant and anti-inflammatory properties have made it well recognized as a therapeutic agent ( Al-Sereiti, Abu-Amer, & Sen, 1999). Furthermore, the neuroprotective effects of RA can be associated with its power to transmogrifying some intracellular cascade events participating in neuronal death ( Fallarini et al., 2009). RA has indicated long-standing benefits for neuronal function, probably due to its ability to overcome the inflammatory response ( Luan, Kan, Xu, Lv, & Jiang, 2013) and decrease the expression of proinflammatory molecules ( Gamaro et al., 2011).

According to previous studies, compounds like RA could reduce DNA damage through their scavenging ability. It suggests a neuroprotective effect for this compound, which can prevent and manage various neurological disorders, like epilepsy. In our previous study, we argued that RA pretreatment could attenuate seizure and oxidative stress, augment the activity of defensive systems, and prevent hippocampal neuronal loss and Mossy Fiber Sprouting (MFS).

The present study aimed to assess the effect of RA on apoptosis, nNOS-positive neurons number, and COX-2 and MAPK immunoreactivity, following intrahippocampal KA in rats.

## 2. Methods

All experiments were performed on adult male Wistar rats (250–300g; N=30). They were housed three to four per cage in a temperature-controlled colony room under light/dark cycle with food and water available ad libitum. Procedures involving animals were conducted in conformity with the National Institutes of Health (NIH) guidelines for the care and use of laboratory animals. In this study, all efforts were made to minimize the number of animals and their suffering.

The rats were randomly assigned to three groups of sham, KA, and KA+RA. For intrahippocampal injections, the rats were anesthetized with chloral hydrate [350 mg/kg; Intraperitoneal (IP)] and placed into the stereotaxic frame (Stoelting Co., USA) with the incisor bar set at 3.3 mm below the interaural line. The dorsal surface of the skull was exposed. Then, a burr hole was drilled using the following coordinates according to the stereotaxic atlas of Paxinos and Watson ( Paxinos G, 1986) with the bregma point as the reference: anteroposterior; 4.1, mm lateral; 4.1 mm, and ventral to the dura; 4 mm. Freshly prepared KA solution (Sigma-Aldrich, USA) (5 μL of normal saline containing 4 μg of kainic acid) was injected into the right side of the hippocampus at a rate of 1 μL/min using a Hamilton microsyringe. The syringe was slowly withdrawn and the rat scalp was sutured. The sham group received the same volume of normal saline. RA (Sigma, USA) at a dose of 10 mg/kg/day was dissolved in propylene glycol and gavaged for one week before surgery ( Karthikkumar, Sivagami, Vinothkumar, Rajkumar, & Nalini, 2012), and the last treatment occurred 1h pre-surgery.

The animals were anesthetized with ketamine, per-fused for 5–8 min with normal heparinized saline, and for 30 min with 4% paraformaldehyde in 0.1 M phosphate buffer (pH 7.4). The rats’ brain was then removed and postfixed overnight in 4% paraformaldehyde at 4°C, then immersed in 30% sucrose phosphate buffer at 4°C.

The hippocampal blocks were prepared; sections were cut at a thickness of 20 μm on a freezing microtome (Leica, Germany). The sections were washed with Phosphate Buffer Saline (PBS). Then, we performed an H2O2 treatment for 10 min. Next, after permeabilization with 0.4% Triton X-100/PBS for 15 min, nonspecific staining was blocked by incubation with 10% normal goat serum in PBS for 1h at room temperature. Accordingly, the sections were incubated with an anti-MAPK primary antibody, anti-COX-2 primary antibody, and anti-nNOS primary antibody (Abcam, USA) at a dilution of 1/500 in a moist atmosphere at room temperature overnight. After that, slides were washed in PBS and incubated for 2h with goat anti-rabbit antibody conjugated with Horseradish Peroxidase (HRP) (Abcam, USA) at a dilution of 1/500 in PBS. Following several rinses in PBS, slides were incubated with 3, 3′-diaminobenzidine (Sigma-Aldrich, Germany) and PBS for 5–10min in the darkness. Slides were then washed, counterstained with 0.1% Cresyl violet, dehydrated in a graded series of alcohol, cleared in xylene, coverslipped with entellan, and microscopically analyzed.

To detect Deoxyribonucleic Acid (DNA) fragmentation and apoptotic cell death, a Terminal Deoxynucleotidyl Transferase (TdT) dUTP Nick-End Labeling (TUNEL) (TUNEL) assay was performed using the In Situ cell death detection kit (Roche, Germany). In this assay, the sections were incubated with proteinase K, rinsed, incubated in 3% H2O2, permeabilized with 0.5% Triton X-100, and consequently rinsed and incubated in the TUNEL reaction mixture. The sections were rinsed and visualized using a converter-POD and subsequent incubation with DAB (3, 3′-diaminobenzidine tetrachloride) and H2O2, counterstained with hematoxylin, then dehydrated and coverslipped and evaluated. A dark brown color indicating DNA breaks was developed. The TUNEL reactivity density was assessed in ≥10 islets for each rat, and its average rate was considered as the final value.

All values were presented as Meam±SEM scores. For morphometric and densitometric analysis, ≥5 sections for each histochemical reaction were used, and UTHSCSA ImageTool, v. 3, 2002 was applied. Regarding immunoreactivity intensity, the following scoring scale was used: no reactivity 0, very mild reactivity 1, mild reactivity 2, moderate reactivity 3, strong reactivity 4, and very strong reactivity 5. All counting and assessments were conducted blind to the treatments received. The obtained data were analyzed using repeated measures and one-way Analysis of Variance (ANOVA) followed by Tukey posthoc test. P<0.05 was considered statistically significant.

## 3. Results

**Figure 1.**
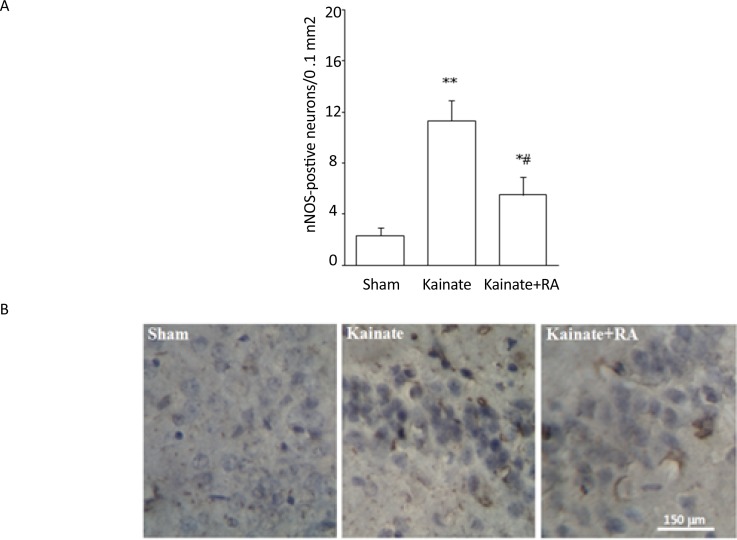
A. The number of nNOS-positive neurons/0.1 mm^2^; and B. The photomicrograph of the CA3 region shown through immunohistochemical staining in the sham, KA, and KA+RA groups RA: Rosmarinic Acid. ^*^vs Sham; P<0.05; ^**^vs Sham; P<0.01; and # vs KA; P<0.05 (Meam±SEM)

**Figure 2.**
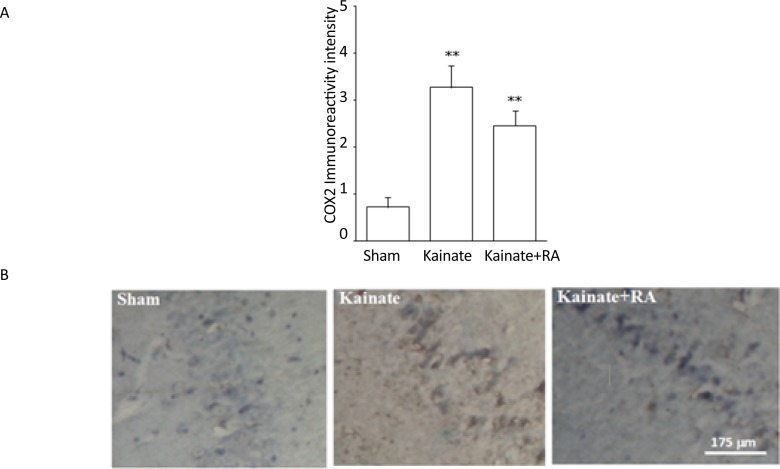
A: COX2 immunoreactivity intensity; and B: The photomicrograph of the CA3 region shown through immunohisto-chemical staining in the sham, KA, and KA+RA groups RA: Rosmarinic Acid; ^**^ vs. Sham; P<0.01(Meam±SEM)

**Figure 3.**
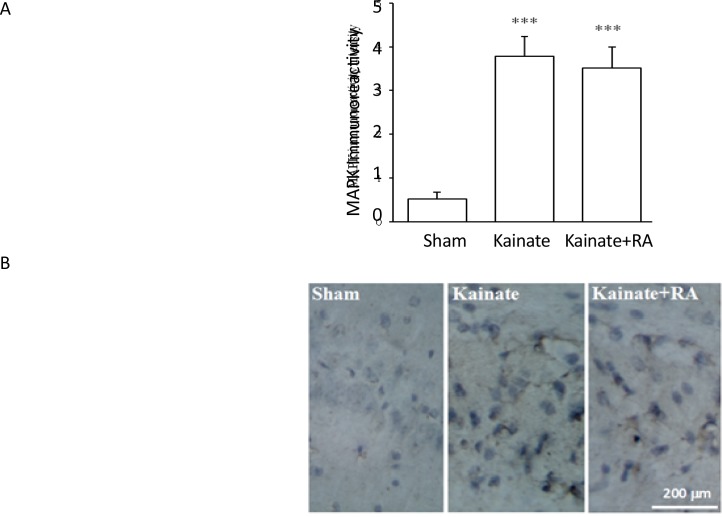
A. MAPK immunoreactivity intensity; and B: The photomicrograph of the CA3 region shown through immunohistochemical staining in the sham, KA, and KA+RA groups RA: Rosmarinic Acid. ^***^ vs. Sham; P<0.001(Meam±SEM)

The immunohistochemical results suggested that KA group had a significantly higher number of nNOS-positive neurons (P<0.01) (Figure 1) and a significant higher reactivity for COX-2 (P<0.01) (Figure 2) and MAPK (P<0.005) (Figure 3), compared to the sham group. In addition, regarding nNOS, KA+RA groups, only 58.2% of the studies rats indicated a significant elevation of the number of nNOS-positive neurons (P<0.05), compared to the sham group. Besides, 51.2% of the studied rats demonstrated a significantly lower number of nNOS-positive neurons, compared to the KA Group (P<0.05) (Figure 1).

Furthermore, the KA+RA group’s COX-2 immuno-reactivity intensity significantly changed, compared to the sham group (P<0.01). In this regard, using RA as a pretreated could reduce approximately 24.7% of COX-2 immunoreactivity intensity, compared with the KA. Additionally, there was no significant difference between the KA and KA+RA groups (Figure 2). Moreover, MAPK immunoreactivity was significantly higher in all of the KA groups (P<0.005) (Figure 3) compared to the sham group without the beneficial effect of RA.

**Figure 4.**
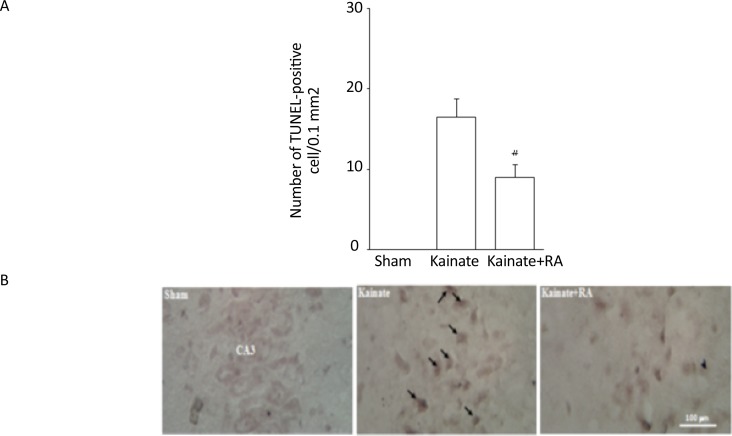
A. The number of TUNEL-positive cell/0.1 mm^2^; and B: The photomicrograph of the CA3 region through showing apoptosis assay in the sham, KA, and KA + RA groups RA: Rosmarinic Acid; ^#^ vs. KA; P<0.05(Mean±SEM)

Determining apoptosis by the TUNEL method indicated that the KA group had a high number of TUNEL-positive neurons apoptotic index, compared to the sham and RA; pretreatment significantly attenuated this effect in these groups, compared to the KA group (P<0.05). Thus, in the KA+RA group, the death rate of neurons decreased to 42.4%. These findings indicated that RA has neuroprotective effects, compared to KA (Figure 4).

## 4. Discussion

The KA-induced seizure model is widely used as a standard model of human temporal lobe epilepsy ( Kiasalari, Roghani, Khalili, Rahmati, & Baluchnejadmojarad, 2013; Tchekalarova et al., 2013). As a structural analog of glutamate, KA activates excitatory amino acid receptors and triggers neuronal membrane depolarization and increases calcium influx through voltage-dependent calcium channel opened by membrane depolarization. This process occurs following the activation of KA receptors with the subsequent induction of the formation of Reactive Oxygen Species (ROS), leading to enhanced oxidative stress ( Kanada et al., 2005). In turn, the increased ROS generation leads to the dysfunction of mitochondrial respiratory chain and damage to the cell structures, subsequently resulting in neuronal damage ( Shih, Chein, Wang, & Fu, 2004).

The brain has an array of antioxidant defensive systems, such as catalase, superoxide dismutase, and reduced Glutathione (GSH), which prevent over-oxidative damages ( Ciftci, Oztanir, & Cetin, 2014). Furthermore, oxidative glutamate toxicity is initiated by high concentrations of extracellular glutamate that prevent cysteine uptake into cells, followed by the depletion of intracellular cysteine and GSH loss.

With a diminishing supply of GSH, there is an accumulation of excessive amounts of ROS and ultimately cell death. Evidence suggested that KA-induced neuronal damage resulted from free radicals ( Han et al., 2012). Additionally, KA enhances MAPK and COX-2 expression ( Hsieh et al., 2011). COX-2 is the primary isoform of cyclooxygenase in the brain, leading to enhanced oxidative stress along with the production of prostaglandins, which may have many injurious effects. COX-2 could also contribute to certain inflammatory responses, neuronal hyperexcitability, and death ( Zhang, Sun, Lei, Yang, & Liu, 2008). Such alterations also occurred in our study. Furthermore, nNOS up-regulation is responsible for neuronal apoptosis and damage that may be responsible for enhanced apoptosis in our study.

Part of the beneficial effect of RA could be attributed to its neuroprotective potential ( Fallarini et al., 2009). In this regard, RA could protect N2A cells against oxidative damage ( Ghaffari et al., 2014). Furthermore, it is a strong protective agent against the 6-hydroxydopamine-induced degeneration of the nigrostriatal dopaminergic system via regulating the ratio of Bcl-2/Bax gene expression, i.e., involved in the apoptotic pathway ( Wang et al., 2012). RA could reduce the detrimental action of neurotoxins and excitotoxic agents, like KA on neurons, thus, limiting the accumulation of extracellular glutamate and preventing the apoptotic death of neurons. The anti-apoptotic potential of RA may also be involved in its beneficial effect.

Previous investigations have suggested that in KA-induced epileptic seizure model, the protective protein Bcl-2 is down-regulated, and apoptosis occurs, consequently ( Zhang, Yan, Wu, Li, & Zhang, 2011). Besides, RA treatment could inhibit the apoptotic cascade by increasing Bcl-2 expression ( Wang et al., 2012). Accordingly, RA could prevent Kainic acid-induced apoptotic cell death. Besides, RA exerts a protective effect on astrocytes, as demonstrated by their increased viability and decreased apoptosis rate induced by H2O2. This process occurs through increasing mitochondrial membrane potential and inhibiting caspase-3 activity and attenuating cellular oxidative stress ( Gao, Wei, Zhao, Xiao, & Zheng, 2005).

KA-induced epilepsy also accompanies inflammation with the increased production of certain prostaglandin, like prostaglandin E2 following enhancement in the mRNA levels of cyclooxygenase and prostaglandin E2 synthase in the brain tissue; anti-inflammatory agents could reduce the condition’s severity ( Ciceri et al., 2002). Besides, KA-induced excitotoxicity through the induction of matrix metalloproteinases leads to selective neuronal death and neuroinflammation in the hippocampus. Furthermore, the inhibitors of such enzymes could attenuate the ensuing neuronal damage; this could be therapeutically relevant in related neurological disorders ( Jourquin et al., 2003). RA could exert anti-inflammatory effects via the attenuation of the expression of nuclear factor-kappaB and tumor necrosis factor-α. Furthermore, RA decreased phosphorylated p53 and the expression of caspase-3 in the kidney; thus, it exhibits anti-apoptotic activity ( Domitrovic, Potocnjak, Crncevic-Orlic, & Skoda, 2014).

## 5. Conclusion

The obtained data suggested that RA exerts a neuroprotective effect against KA-induced injury; it may be mediated through the downregulation of nNOS and reduction of apoptosis.
